# Precise assessment of facial skin based on multimodal data fusion of the microbiome

**DOI:** 10.1002/qub2.70046

**Published:** 2026-06-23

**Authors:** Fan Meng, Junhui Zhang, Chunying Yuan, Ruichen Li, Yangyang Sun, Hong Jiang, Suzhen Yang, Yan Li, Xiaoquan Su

**Affiliations:** ^1^ College of Computer Science and Technology Qingdao University Qingdao China; ^2^ Research & Innovation Center Shandong Freda Biotech Co., Ltd Jinan China

**Keywords:** bioinformatics, deep learning, facial skin, microbiome, multi‐modal fusion

## Abstract

The skin microecology plays a vital role in maintaining cutaneous health and is intricately linked to host skin phenotypes. However, there remains a lack of precise and quantitative biomarkers for evaluating skin health conditions, making it challenging to identify individuals at potential risk of microecological imbalance. In this study, we collected facial skin microbiomes from 242 female volunteers aged 16–50 years in Shanghai, China. Microbial communities were surveyed using 16S rRNA gene sequencing and integrated with high‐resolution facial images and host skin phenotypes for comprehensive analysis. Our findings reveal that although the community complexity of the facial microbiome is comparable between individuals with “ideal” and “non‐ideal” skin conditions, the composition and structure of key microbes varied significantly between the two groups. Differential abundance analysis further emphasized the role of interactions between the skin microbiome and host phenotypic traits in skin aging and status transitions. To enable a more holistic and quantitative assessment of facial skin status, we developed a novel multimodal skin index (MSI) that fuses multiple modal data from facial images, microbiome profiles, and host skin phenotype features through a deep learning‐based framework. Importantly, MSI identified individuals with an outwardly healthy facial appearance but significant underlying microbial dysbiosis that conventional diagnostic approaches often overlook. This work enables the detection of such hidden risks, offering new avenues for individualized facial skin health assessment, precision dermatology, and microbiome‐informed esthetic interventions.

## 1 INTRODUCTION

The skin is the body’s largest organ and serves as the primary barrier against environmental stressors and psychological factors. Among all skin regions, the facial skin is the most directly exposed to external conditions and exhibits high sensitivity and structural complexity, making it a valuable model for studying cutaneous health and disease. Compared to other body sites, facial skin possesses distinct physiological characteristics [1], including a higher density of sebaceous glands, particularly in the T‐zone (forehead, nose, and chin). This increased sebaceous activity enhances sebum secretion, which contributes to skin hydration, protection, and barrier function [2]. Multiple factors influence facial skin health, encompassing both physiological and molecular attributes—such as moisture level, pH, and lipid content [3, 4]—as well as image‐derived phenotypes including brightness, wrinkle formation, texture, and pore visibility [5, 6, 7, 8, 9]. In addition, host‐related variables such as gender, age [10], occupation, hygiene habits, skincare and medication use, and diet significantly contribute to interindividual variation [11, 12].

The facial skin also harbors a dense and diverse microbial ecosystem that plays a crucial role in maintaining skin homeostasis. These microorganisms contribute to protective functions such as barrier reinforcement, pathogen inhibition, immune modulation, and bioactive metabolite production [13, 14, 15, 16]. Consequently, the skin microbiome has attracted growing interest for its potential in health monitoring, disease prevention, and personalized skincare applications [17]. For example, a recent study utilizing 16S rRNA gene sequencing found that an increased abundance of bacterial taxa such as *g__Propionibacterium*, *g__Corynebacterium*, and *g__Bacillus* during skincare routines was associated with improved skin texture [18]. However, despite advances in microbiome research, the interplay between skin microbial communities and image‐derived facial features remains insufficiently explored. Moreover, comprehensive methodologies for the objective and integrative assessment of skin condition are still lacking.

In this study, we recruited a cohort of 242 female participants in Shanghai, China, comprising both “ideal” and “non‐ideal” facial skin phenotype groups (Table 1). Using 16S rRNA amplicon sequencing, we profiled the facial microbiome and performed bioinformatics analyses to investigate the role of bacterial communities in determining ideal skin (IS) conditions. By fusing multimodal facial data, including microbiome, host skin phenotype, and facial image characteristics, through a deep learning model, we proposed a new approach for comprehensive and precise skin condition evaluation (Figure 1). Moreover, this model enables effective prediction of risks underlying skin dysbiosis, especially for individuals who may otherwise be overlooked by traditional dermatological or cosmetic assessments. By overcoming limitations of conventional evaluation methods, our approach marks a significant advancement in computational skin health assessment and contributes to the growing field of microbiome‐informed personalized dermatology.

**TABLE 1:** Introduction to dataset information.

Group	Number of samples	Age	Total number of reads
Ideal skin (IS)	53	18–35	2,349,977
Non‐ideal skin (non‐IS)	189	16–50	8,408,688

**FIGURE 1:**
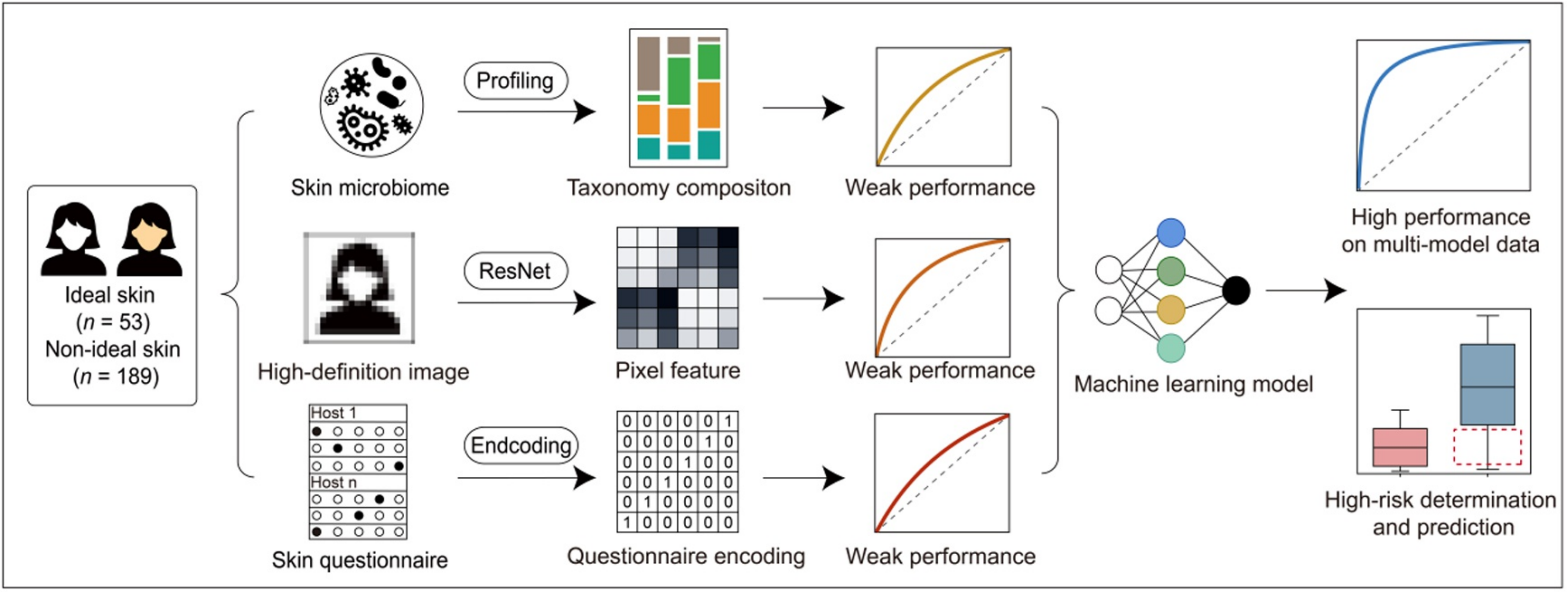
Overall workflow for multimodal skin condition evaluation. Single data modalities, including microbial profiles, host phenotypic data, or images, showed limited predictive performance when used alone. By integrating these modalities through a deep learning framework, the predictive power was significantly improved, enabling more accurate assessment of skin health and identification of high‐risk conditions.

## 2 RESULTS

### 2.1 Cohort design

This study recruited a total of 242 healthy female volunteers aged 16–50 years in Shanghai, China (Table 1; refer to Supporting Information S1: Table S1 for details). Using a standardized evaluation framework (refer to the MATERIALS AND METHODS section for details), facial skin conditions were quantitatively assessed across four dimensions: acne, pores, pigmentation, and wrinkles. Based on these scores, participants were categorized into two groups: the ideal skin group (IS, *n* = 53) and the non‐ideal skin (non‐IS) group (non‐IS, *n* = 189) [19]. To characterize the facial skin microbiome, 16S rRNA gene amplicon sequencing was performed on cheek swab samples from all participants. We also collected a wide range of phenotypic data, including demographic variables (e.g., age, body mass index), skin physiological parameters (e.g., skin type, VISIA imaging data), clinical information (e.g., disease history), and behavioral factors (e.g., frequency of cosmetic product use), ensuring comprehensive and accurate characterization. Among these features, skin physiological parameters were obtained using the VISIA‐CR imaging system (Canfield Imaging Systems) and analyzed quantitatively in a standardized manner. Details of the experimental design, cohort stratification, and sequencing protocols are provided in the MATERIALS AND METHODS section.

### 2.2 The subtle association between the skin microbiome and skin health

Previous studies have found that numerous skin disorders and the natural aging process are often accompanied by substantial shifts in microbial community structure [20]. For instance, in atopic dermatitis, dynamic changes in the microbiome have been well documented, notably characterized by an overgrowth of *s__Staphylococcus_aureus* and a reduction in *s__Staphylococcus_epidermidis* [21]. However, there remains a limited understanding of how microbial community structure varies between IS and non‐IS states in association with dynamics in multiple features.

In this study, we first examined microbiome alterations under different skin conditions. Alpha diversity analysis revealed that although there was a trend toward different Shannon index distributions between the ideal and non‐ideal groups, this difference was not statistically significant (Wilcoxon test *p* > 0.05) (Figure 2A). Among the total of 40 microbial genera detected, 13 were retained after marker screening (refer to the MATERIALS AND METHODS section for details), which modestly improved the distinction between groups, although the differences remained statistically insignificant. Spearman correlation analysis showed that Shannon diversity was significantly but weakly negatively correlated with both transepidermal water loss (TEWL) (*r* = −0.207, *p* < 0.01) and sebum levels (*r* = −0.258, *p* < 0.01) (Figure 2B), suggesting a potential link between microbiome diversity and skin barrier function.

**FIGURE 2:**
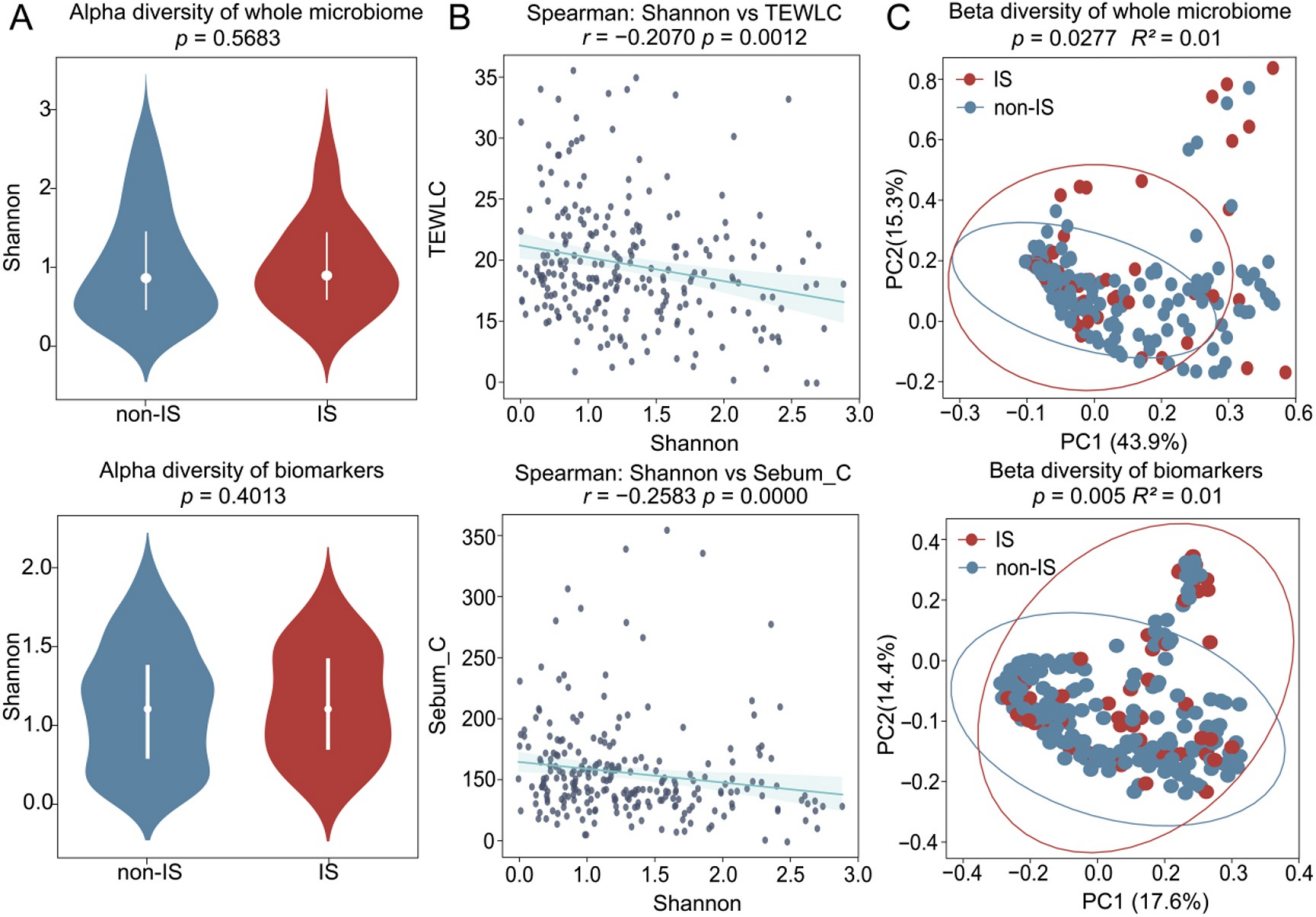
Analysis of microbiome diversity. (A) Distribution of alpha diversity of all microbes and screened biomarkers in the IS and non‐IS groups. (B) Spearman correlation analysis results of the Shannon index of the microbiome and skin physiological parameters. (C) Principal component analysis diagrams of the beta diversity of all microbes and the screened biomarkers between the IS and non‐IS groups. Source data are available in Supplementary Datasheets 1–3. IS, ideal skin; non‐IS, non‐ideal skin; PC, principal component; TEWLC, transepidermal water loss capacity.

Beta diversity analysis based on Bray–Curtis dissimilarity indicated partial spatial overlap between samples from the two groups. Although some compositional differences were observed, they did not reach statistical significance prior to feature selection (Figure 2C). However, after applying feature‐based screening, the intergroup differences became more pronounced and statistically significant (PERMANOVA test *p* < 0.01), suggesting that discriminatory microbial features are more likely associated with skin condition‐related taxonomic composition or functional potential, such as antimicrobial peptide production or immune modulation, rather than with overall diversity alone.

### 2.3 Integrated modeling of microbiome and host phenotypic variables

To enable accurate assessment of skin condition, we constructed a data model using the XGBoost (extreme gradient boosting) algorithm based on the relative abundance of all microbes [21]. Model performance was evaluated using the area under the receiver operating characteristic curve (AUC) via fivefold cross‐validation (refer to the MATERIALS AND METHODS section for details). The solely microbiome‐based model demonstrated limited classification power, achieving an AUC of only 0.62 (Figure 3A). This suboptimal performance may be attributed to the lack of significant differences in alpha diversity between the IS and non‐IS groups (Figure 2A), suggesting that microbial composition alone may be insufficient to fully distinguish skin health status.

**FIGURE 3:**
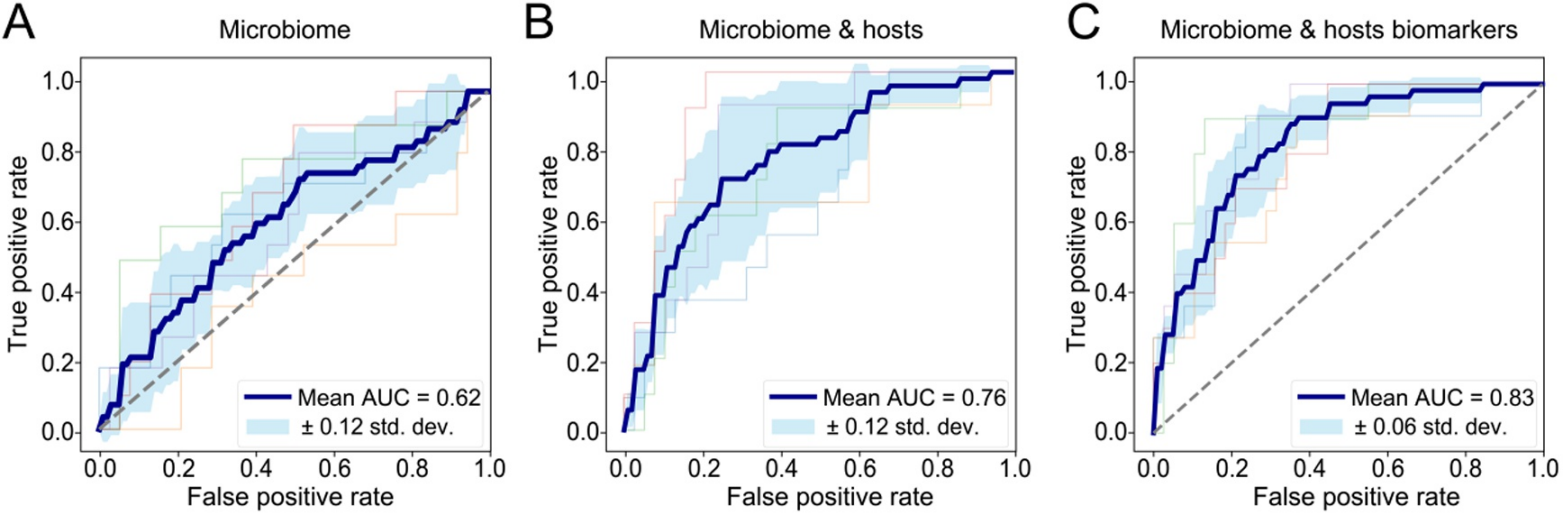
ROC curves of different models and their combinations. (A) The initial model was constructed based on all genus information. (B) The model was enhanced by integrating all features of the microbiome and host phenotypic data. (C) The model performance was further enhanced by refining and noise cancellation of the microbiome and the host phenotypic features. Source data are available in Supplementary Datasheet 4. AUC, area under the receiver operating characteristic curve; dev, deviation; ROC, receiver operating characteristic; std, standard.

Considering that skin condition is influenced not only by the microbiome but also by host phenotypic traits and lifestyle factors [22], we further developed an integrated, multimodal fusion model. Host phenotypic and lifestyle variables—including age, sex, and dietary habits—were encoded using one‐hot encoding and fused with microbiome features to form a composite feature matrix.

This multimodal fusion model showed significantly improved performance, with the AUC increasing to 0.76 (Figure 3B). After applying feature selection to reduce noise and enhance model interpretability, the AUC further increased to 0.83 (Figure 3C). These findings underscore the critical role of host phenotypic information in enhancing prediction accuracy and demonstrate the potential of multidimensional feature integration for future research in skin health assessment and personalized dermatology.

### 2.4 Correlation analysis between bacterial flora information and host phenotypic characteristics

To investigate the relationship between microbial communities and host phenotypic characteristics, we performed a systematic assessment of intergroup variation in bacterial genera after feature selection using analysis of variance (ANOVA). The results revealed that multiple genera exhibited significant differences under distinct host conditions, with several taxa demonstrating strong statistical significance (ANOVA test *p* < 0.01) (Figure 4).

**FIGURE 4:**
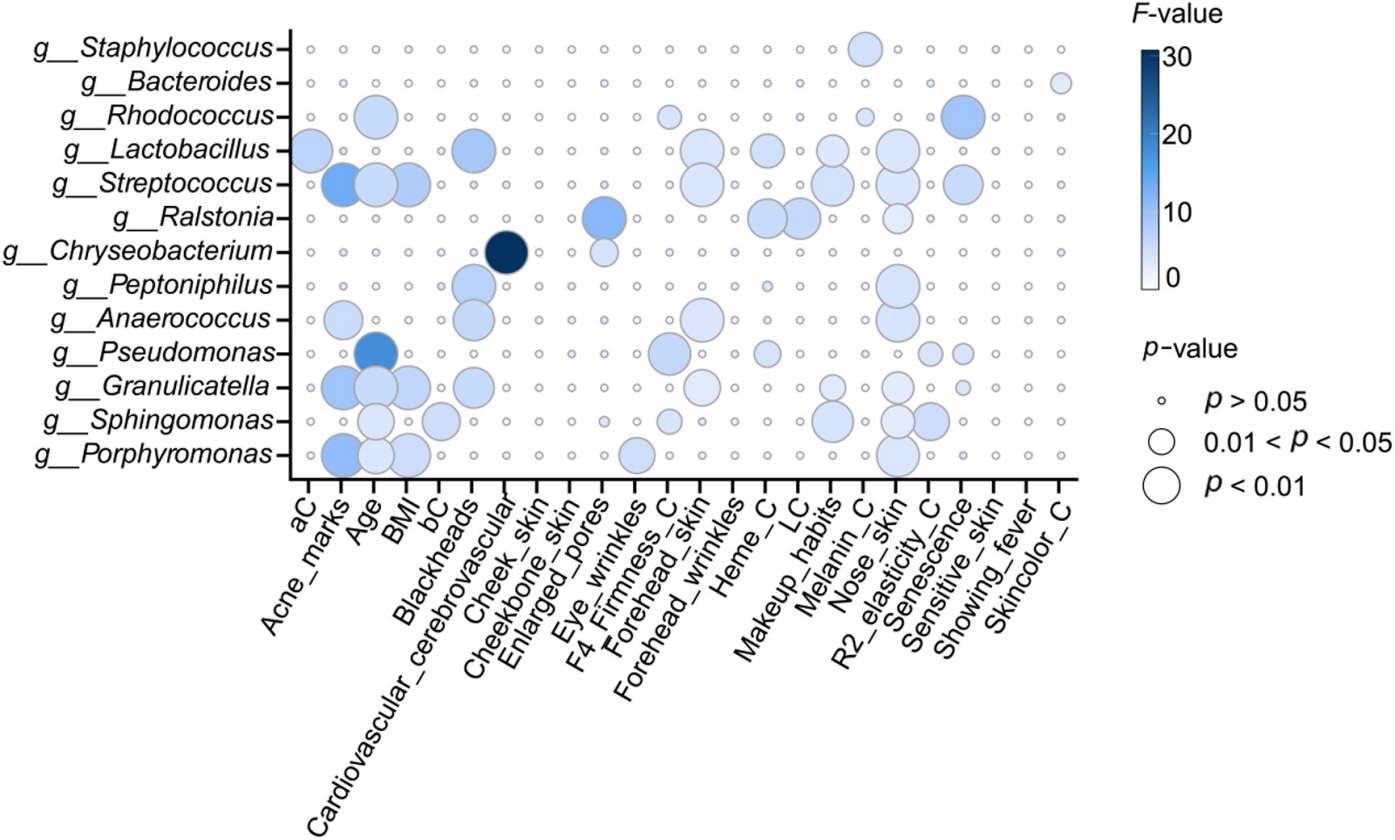
ANOVA correlation analysis between microbes and host phenotypes. ANOVA statistical analysis of microbial genus information and host phenotypic variables. Source data are available in Supplementary Datasheet 5. aC, a‐axis chroma; ANOVA, analysis of variance; bC, b‐axis chroma; BMI, body mass index; C, chroma; LC, luminance chroma.

In the microbiome–phenotype association analysis for skin health indicators, *g__Streptococcus* showed a significant positive correlation with the severity of acne marks (ANOVA test *F* = 13.57, *p* < 0.01), consistent with previous reports on its enrichment in acne‐prone skin [23]. This genus may aggravate inflammatory and pigmentation responses by activating innate immunity. The relative abundance of *g__Ralstonia* was significantly elevated in participants with enlarged pores (ANOVA test *F* = 11.59, *p* < 0.01), supporting the hypothesis that *g__Ralstonia* may influence sebum metabolism and keratinization [24]. Similarly, *g__Lactobacillus* was significantly associated with blackhead formation (ANOVA test *F* = 8.91, *p* < 0.01), aligning with the “microbial–sebum oxidation” hypothesis [25], which posits that *g__Lactobacillus* may contribute to acne development via modulation of sebum oxidation or interspecies microbial competition.

Notably, *g__Porphyromonas* was significantly enriched in individuals with prominent acne marks (ANOVA test *F* = 10.80, *p* < 0.01), corroborating earlier studies implicating this genus in postinflammatory hyperpigmentation through the promotion of localized inflammation [26, 27]. In addition, *g__Pseudomonas* exhibited the most pronounced variation (ANOVA test *F* = 18.07, *p* < 0.01), highlighting its potential role in maintaining skin microbial homeostasis and supporting its relevance in the dynamic regulation of skin barrier integrity and microbial succession [28].

Together, these findings underscore the close associations between specific microbial taxa and host skin phenotypes, providing new evidence for the role of the skin microbiome in conditions such as acne, enlarged pores, and pigmentation. This study supports the microbiome–skin interaction paradigm, offering a scientific foundation for the development of microbiome‐informed personalized skincare strategies.

### 2.5 Innovative application of multimodal fusion model and IS indicators

To further improve the accuracy of skin condition assessment, we systematically integrated facial image data, microbiome profiles, and host phenotypic features enhanced by deep learning‐based feature extraction and encoding to develop a novel multimodal fusion model (refer to the MATERIALS AND METHODS section for details). This resulting model exhibited a substantial performance gain on an independent test set, achieving an AUC of 0.91 (Figure 5A,B), which markedly outperformed both single‐modal models (e.g., the microbiome model, AUC = 0.62) (Figures 3A and 5A) and bimodal models (e.g., the microbiome and host biomarkers model, AUC = 0.83) (Figure 3C). This improvement underscores the advantage of multimodal data fusion in capturing the complex biological and visual signatures associated with skin condition.

**FIGURE 5:**
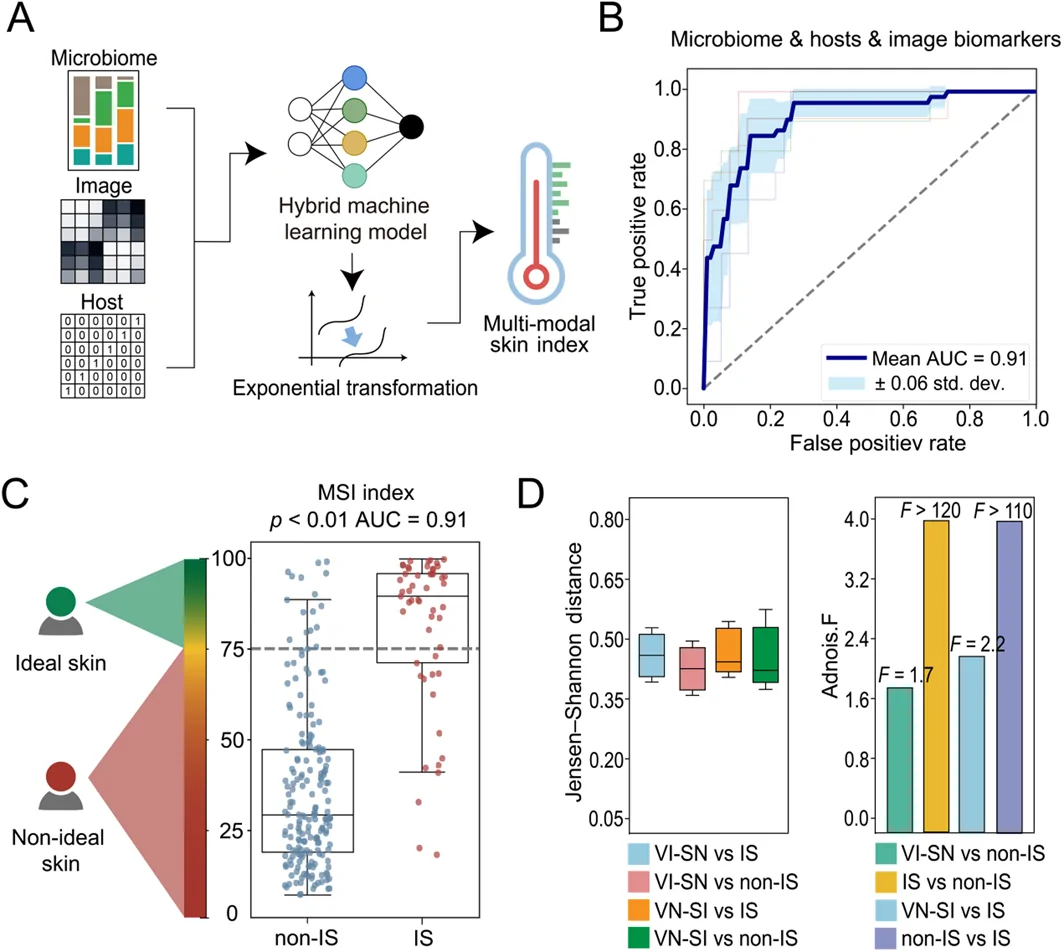
The development of three‐modal models and MSI. (A) Schematic diagram of the MSI based on an integrated multimodal data modeling. (B) ROC curve of the comprehensive model integrating image features, microbial features, and host phenotypic features. (C) Distribution of the MSI between the IS group and the non‐IS group. (D) Analysis of microbiome structural similarity among MSI‐identified subgroups. Source data are available in Supporting Information S2: Datasheet 6. AUC, area under the receiver operating characteristic curve; IS, ideal skin; MSI, multimodal skin index; ROC, receiver operating characteristic; VI‐SN, individuals with visual non‐ideal but ideal MSI scores; VN‐SI, individuals with visual ideal but non‐ideal MSI scores.

Building upon this framework, we further proposed the multimodal skin index (MSI)—an innovative, quantitative scoring system derived from the multimodal model (Figure 5A). The MSI ranges from 0 to 100, with higher scores reflecting skin conditions closer to the ideal state. Based on MSI scores, participants were stratified into two categories: IS group (MSI ≥ 75) and non‐IS (MSI < 75). Validation analyses demonstrated that MSI scores were significantly correlated with clinical skin assessment outcomes (ANOVA test *p* < 0.01) (Figure 5C), and the MSI‐based classification yielded high discriminatory power (AUC = 0.91), confirming the robustness and predictive accuracy of this index in skin condition evaluation.

### 2.6 Developmental trends and risk prediction of skin condition by MSI

Interestingly, visual assessment alone sometimes misclassified individuals as having an IS condition. However, when evaluated using the MSI, some of these individuals were reclassified as non‐ideal (designated as VI‐SN). Conversely, another subset of individuals displayed average or non‐IS appearances but were classified as ideal by the MSI model (designated as VN‐SI).

Within our experimental cohort, VI‐SN individuals visually resembled those in the IS group, but their microbiome profiles closely matched those of the non‐IS group. As a result, they were classified as non‐ideal by the MSI model. Microbial distance analysis further supported such classifications. The Jensen–Shannon divergence between VI‐SN and non‐IS microbiomes was low, indicating strong similarity. This was corroborated by Adonis analysis, which yielded low *F*‐values when comparing VI‐SN to non‐IS (Figure 5D), reinforcing their microbiological resemblance.

In contrast, VN‐SI individuals, though lacking ideal visual features, exhibited microbiome compositions similar to those in the IS group and were thus recognized as ideal by the MSI. For VN‐SI individuals, although the Jensen–Shannon distance to both IS and non‐IS groups was comparable, Adonis analysis showed significantly higher *F*‐values when comparing VN‐SI to non‐IS than when comparing VN‐SI to IS. This suggests that the VN‐SI microbiome is more aligned with the ideal microbiome state.

In summary, these findings reveal that visually IS may mask underlying microbiome imbalances, highlighting the limitations of surface‐level assessment. The MSI effectively identifies such “high‐risk” individuals (VI‐SN) and also detects “potential ideal” individuals (VN‐SI). Thus, the MSI model offers a powerful tool for early risk detection and personalized skin health monitoring, enabling proactive intervention before clinical symptoms become apparent.

## 3 DISCUSSION AND CONCLUSION

The scientific assessment of skin health remains a critical focus within contemporary biomedical research. In this study, we employed a multimodal integrative analysis strategy, combining biomarker screening, feature extraction and encoding, and deep learning modeling to develop a comprehensive skin condition assessment system. This system not only enables precise prediction of skin health status but also introduces the MSI—an innovative quantitative metric that integrates multidimensional features. MSI is defined as a continuous score, with 75 used as a reference threshold that is aligned with the decision criterion of the underlying binary classification model through a power transformation. Therefore, MSI should be interpreted primarily as a continuous indicator of skin status rather than a fixed diagnostic boundary. Given potential differences in demographic characteristics and skin‐type distributions across populations, its application to other cohorts may require recalibration of the transformation function and threshold. Overall, the MSI provides a quantitative framework for evaluating skin conditions and identifying potential high‐risk groups, offering a novel tool for the quantitative assessment of skin health. Our multimodal approach substantially enhances the comprehensiveness and accuracy of skin condition evaluation, thereby providing a scientific foundation for personalized skincare regimen formulation.

Initially, we characterized the skin microbiome via 16S rRNA gene sequencing. Results revealed significant differences in beta diversity of microbial communities between the IS and non‐IS groups, primarily reflected in differences in specific species composition and functional potential rather than in alpha diversity metrics. Specifically, the Shannon diversity index was weakly but significantly negatively correlated with both TEWL and sebum secretion, suggesting that higher microbial diversity may modestly suppress sebum production and enhance barrier function. Nevertheless, the modest effect size observed indicates that overall community diversity is unlikely to be the dominant determinant of skin condition; rather, specific bacterial genera or functional genes warrant deeper investigation.

Although the skin microbiome reflects upstream functional states of the cutaneous ecosystem, it provides only a partial perspective. Integrating additional modalities offers complementary information: Image features capture downstream manifestations such as inflammation, lipid metabolism, and barrier dysfunction, whereas host characteristics provide exogenous contextual factors that influence both microbial composition and skin phenotype. Although the deep features extracted by ResNet‐18 (deep residual network) cannot be directly mapped onto specific physical quantities, previous studies have demonstrated that convolutional neural networks hierarchically encode visual patterns ranging from low‐level edges and textures to higher‐level structural representations. In the context of facial skin images, these features are therefore expected to primarily reflect visual attributes such as skin texture, pigmentation distribution, and pore‐related patterns. Together, these three modalities form a coherent causal chain—host background, microbial functional changes, and skin phenotype—enabling multimodal models to leverage complementary signals and learn cross‐modal interactions. By integrating microbial, host phenotypic, and facial image features through biomarker selection and multimodal fusion, we constructed a robust machine learning model for skin condition prediction. Notably, during model validation, we identified two distinct phenotypic subgroups that showed discordant predictions between the single‐modal and multimodal models. This discrepancy may reflect underlying biological heterogeneity in microbiome–skin interactions and suggests that the multimodal framework captures complementary information beyond any single data source. Importantly, these subgroups may represent individuals with latent skin health risks who are not readily detectable by conventional single‐modality assessments, thereby highlighting the potential of the proposed model for early risk stratification and personalized skin management.

Although this study advances understanding of the skin microbiome’s role in skin health, limitations remain. First, the study cohort consisted exclusively of female participants (aged 16–50 years) from a single city, with predominantly Asian skin types, and thus the findings may not be generalizable to males, individuals from other locations, or those with different skin types. Second, because 16S rRNA sequencing provides limited functional resolution, this study can identify associations between certain microbial genera and skin phenotypes but cannot establish causal relationships between the microbiome and skin traits. By expanding the sample population across diverse demographics and incorporating functional metagenomic data, future research is poised to uncover broader patterns in skin health assessment, thereby strengthening the theoretical basis for precision dermatology.

## 4 MATERIALS AND METHODS

### 4.1 Experimental design and sample collection

This study recruited 242 female volunteers aged 16–50 years from Shanghai, China. The inclusion criteria were as follows: (1) no use of oral or injectable antifungal agents or antibiotics within the past 3 months; (2) no use of topical anti‐inflammatory medications on the face within the past 2 months; (3) no history of asthma or other chronic respiratory diseases; and (4) not pregnant or breastfeeding. All participants provided written informed consent prior to enrollment. The study was conducted in accordance with the ethical principles outlined in the Declaration of Helsinki and was approved by the Ethics Committee of the Sixth People’s Hospital Affiliated to Shanghai Jiao Tong University (Approval No. 2021‐KY‐15).

All facial images were collected using a single VISIA‐CR imaging system operated by trained technicians. During acquisition, technicians performed real‐time inspection to ensure image sharpness and the absence of motion blur. A fixed illumination mode, camera position, viewing angle, and subject‐to‐camera distance were used for all participants. A chin rest and positioning frame ensured consistent facial alignment. Although images were captured across several days, all sessions were conducted with the same hardware, software, and illumination settings. VISIA’s integrated lighting and calibration system maintains consistent exposure and color rendering across sessions, preventing date‐related batch effects.

Quantitative evaluations included indicators such as acne, pore size, pigmentation, and wrinkle formation. Additional parameters assessed included facial redness, glossiness, and acne mark distribution. The inclusion criteria for subjects in the IS group were the absence of visible redness, oiliness, dullness, telangiectasia, scaling, dryness, and desquamation, and no large areas of scars, pigmentation, or skin lesions. Comprehensive assessments were performed using multiple clinical scales (refer to Supporting Information S1: Table S2 for details), including the pore scoring system, the Fitzpatrick wrinkle scale, the acne grading system, and the melasma area and severity index. Based on the evaluation results, participants were categorized into the IS group (IS, *n* = 53) and the non‐IS group (non‐IS, *n* = 189).

All participants underwent standardized skin parameter assessments after facial cleansing with water. Frontal and lateral facial images were obtained using the VISIA‐CR imaging system (Canfield Scientific, Fairfield, New Jersey, USA). A series of devices from Courage + Khazaka (CK, Cologne, Germany) were used to evaluate multiple physiological skin parameters: Corneometer CM 825 for skin hydration; Tewameter TM 300 for TEWL; Sebumeter SM 815 for sebum secretion; Skin‐Glossymeter GL 200 for skin glossiness; Mexameter MX 18 for melanin and hemoglobin content; Skin‐pH‐Meter pH 905 for surface pH levels; and Cutometer dual MPA 580 for skin elasticity (R2 parameter). Demographic information and general health status were also recorded as metadata for each participant.

### 4.2 DNA extraction and sequencing

Before sample collection, all subjects were instructed to avoid using any skincare products or cosmetics after facial cleansing on the night before and on the day of testing. Prior to sampling, they were required to sit quietly for 30 min in a temperature‐ and humidity‐controlled environment (21 ± 1°C; relative humidity 50% ± 5%) to stabilize skin conditions. Skin microbiome samples were then collected by repeatedly swabbing a 3 cm × 3 cm area on the left cheek at least 30 times using a sterile cotton swab soaked in a buffer containing 0.9% NaCl and 0.1% Tween‐20. Samples were immediately stored at −80°C until further analysis.

Microbial genomic DNA was extracted using the FastDNA Spin Kit for Soil (MP Biomedicals, USA). DNA integrity, concentration, and purity were assessed via 1% agarose gel electrophoresis (Biowest, Spain) and NanoDrop 2000 Spectrophotometer (Thermo Fisher Scientific, Waltham, MA, USA). The V3–V4 hypervariable regions of the bacterial 16S rRNA gene were amplified using primers 338F (5′‐ACTCCTACGGGAGGCAGCAG‐3′) and 806R (5′‐GGACTACHVGGGTWTCTAAT‐3′) by PCR. PCR products were purified using the AxyPrep DNA Gel Extraction Kit (Axygen Biosciences, Union City, CA, USA). Libraries were constructed with the NEXTFLEX Rapid DNA‐Seq Kit and sequenced on the Illumina MiSeq PE300 platform. A total of 242 sequence samples were generated.

### 4.3 Data preprocessing

This study integrated three complementary data types for a multidimensional analysis of skin condition: (1) facial image data, (2) host phenotypic data, and (3) skin microbiome data. For facial image data, standardized preprocessing was performed, including facial region cropping, image format normalization (scaling RGB values to the [0, 1] range), and resizing to a consistent input shape. A deep learning ResNet‐18 (deep residual network) model [29] pretrained on the ImageNet dataset was then applied for transfer learning to extract image‐based features. The final classification layer of the network was removed, and all convolutional layers were frozen to retain the stability of the feature extractor. The preprocessed image was fed into the model, and a 224‐dimensional feature vector was extracted from the final convolutional layer as the image representation.

For host phenotypic data, all variables were discretized (refer to Supporting Information S1: Table S3 for details). Continuous variables (e.g., age) were transformed into ordered categorical groups based on clinical relevance (e.g., “≤20,” “21–30,” “>30”), whereas nominal categorical variables (e.g., acne grade) retained their original categories. One‐hot encoding was used to convert all variables into binary vectors, ensuring that variables across different dimensions contributed equally to downstream analyses.

The raw 16S rRNA sequencing data were processed using the Parallel‐Meta Suite [30] following a standardized workflow, which sequentially included quality control, chimera removal, paired‐end read merging, sequence denoising, and amplicon sequence variant (ASV) picking. Subsequently, representative sequences of each ASV were aligned against the Greengenes reference database (version 13‐8) with a similarity of 99% for taxonomic annotation.

All three modalities—microbiome relative abundance profiles, image embeddings, and host phenotype vectors—were preprocessed to ensure that each row corresponded to the same individual sample. Following this harmonization step, a late‐fusion strategy was implemented, in which the modality‐specific feature vectors were concatenated along the feature dimension to generate an integrated multimodal representation for downstream analyses.

### 4.4 Diversity and statistical analysis of facial microorganisms

Based on the microbial community composition at the genus level, we first employed the Mann–Whitney *U* test to assess intergroup differences in alpha diversity, measured by the Shannon index. Beta diversity was analyzed using principal coordinate analysis based on the Bray–Curtis distance to visualize differences in microbial composition between groups. Subsequently, 13 key microbial genera were identified through feature importance ranking from the XGBoost model. The alpha and beta diversity analyses were then repeated using only these selected genera to further evaluate their discriminatory power. To investigate associations between the microbiome and host physiological characteristics, Spearman’s rank correlation was performed between the Shannon index and various skin biophysical parameters (e.g., TEWL, sebum content). For categorical host factors (with ≥2 groups), we used permutational multivariate analysis of variance to assess differences in the composition of the selected microbial genera across groups. *F*‐statistics were calculated to quantify the effect size, and *p* < 0.01 was considered statistically significant.

### 4.5 Establishment of multimodal models

Machine learning models were constructed using the XGBoost implementation provided by scikit‐learn [31]. First, models were built using image features as independent variables. Subsequently, microbial features were used as input to construct a separate model. For both types of data, feature importance scores were computed and ranked in descending order. Based on the rankings, features were incrementally added to the model in an iterative manner to optimize performance. The AUC was used as the evaluation metric. Using this approach, 13 genus‐level microbial features were identified as candidate biomarkers for downstream analysis. Host phenotypic variables were also evaluated using the same feature selection strategy, resulting in 24 optimal features. Finally, selected features from the microbiome, host phenotypes, and image data were integrated to form a multimodal dataset. An XGBoost model was then trained on this fused multisource feature set. The hyperparameter scale_pos_weight was enabled during XGBoost training to balance the positive and negative samples. Hyperparameter optimization was conducted using a two‐stage strategy, with RandomizedSearchCV for coarse‐grained exploration followed by GridSearchCV for fine‐grained refinement. In addition, a stratified sampling scheme was employed for data partitioning to ensure that class proportions were preserved between the training and test sets. Through iterative training and evaluation, the optimal combination of microbiome, phenotypic, and image‐derived features was identified, enabling the construction of a robust predictive model for facial skin condition.

### 4.6 Construction of the MSI index

A multimodal prediction model was developed using image, microbiome, and phenotypic data from 242 individuals. A fivefold cross‐validation strategy was applied, in which the data were cyclically partitioned into a 70% training set and a 30% test set. After training, the model generated the predicted probability for each sample belonging to the positive class. These probabilities were transformed using a power function (Equation 1) and mapped to a score ranging from 0 to 100:

(1) $$\text{Score}=100\times p^{\alpha }$$

where p represents the predicted probability for each sample, and the exponent α is derived by mapping the conventional binary classification threshold *p* = 0.5 to a clinically interpretable score of 75. Substituting these values into Equation (1):

(2) $$75=100\times 0.5^{\alpha }$$

(3) $$\alpha =\frac{\log_{e}\left(0.75\right)}{\log_{e}\left(0.5\right)}$$

This transformation in Equations (2) and (3) ensures that the score ranges from 0 to 100 while aligning the classification threshold *p* = 0.5 with the predefined cutoff score of 75. Based on this threshold, samples with a score ≥75 were classified into the IS group (MSI ≥ 75), whereas those with a score <75 were classified into the non‐IS group (MSI < 75).

This index—referred to as the MSI—provides a quantitative evaluation of facial skin condition.

## AUTHOR CONTRIBUTIONS

**Fan Meng**: Formal analysis; methodology; software; validation; writing—original draft. **Junhui Zhang**: Investigation; methodology; visualization. **Chunying Yuan**: Data curation; resources. **Ruichen Li**: Data curation; formal analysis. **Yangyang Sun**: Formal analysis; validation; writing—review and editing. **Hong Jiang**: Data curation; formal analysis. **Suzhen Yang**: Conceptualization; project administration; resources. **Yan Li**: Conceptualization; investigation; project administration; supervision. **Xiaoquan Su**: Conceptualization; funding acquisition; investigation; project administration; supervision; writing—review and editing.

## CONFLICT OF INTEREST STATEMENT

Chunying Yuan, Hong Jiang, Suzhen Yang, and Yan Li are employed by Shandong Freda Biotech Co., Ltd.

## ETHICS STATEMENT

All participants provided informed consent before study participation. The study protocol was conducted in accordance with the principles of the World Medical Association Declaration of Helsinki and relevant biomedical research ethical guidelines. This study was prospectively approved by the Ethics Committee of the Sixth People’s Hospital Affiliated to Shanghai Jiao Tong University (Approval No. 2021‐KY‐15).

## Supporting information

Supporting Information S1

Supporting Information S2

## Data Availability

All source data in this work have been deposited in the Sequence Read Archive under Project ID PRJNA750340. The source code, usage guidelines, and additional resources are available in the GitHub repository (qdu‐bioinfo/MSI). Supplementary data are available in the GitHub repository (qdu‐bioinfo/MSI/tree/main/supplementary), and all source data for figures are accessible in the GitHub repository (qdu‐bioinfo/MSI/tree/main/data). The MSI was developed using the Python‐based PyTorch framework. The source code, usage guidelines, and additional resources are available in the GitHub repository (qdu‐bioinfo/MSI).
